# Multiple Myeloma Patients at Various Cytogenetic Risks Benefit Differently from Autologous Stem Cell Transplantation as a Consolidation Therapy

**DOI:** 10.1155/2015/613045

**Published:** 2015-01-22

**Authors:** Tianmei Zeng, Lili Zhou, Hao Xi, Weijun Fu, Juan Du, Chunyang Zhang, Hua Jiang, Jian Hou

**Affiliations:** Department of Hematology, The Myeloma and Lymphoma Center, Changzheng Hospital, The Second Military Medical University, 415 Fengyang Road, Shanghai 200003, China

## Abstract

*Aim.* To evaluate whether patients with multiple myeloma at various risks can still benefit the same from autologous stem cell transplantation consolidation in the era of novel agents. We retrospectively analyzed 67 consecutive myeloma patients receiving autologous stem cell transplantation after bortezomib and/or thalidomide based inductions. Totally 17 high-risk, 24 intermediate-risk, and 26 low-risk patients were enrolled, based on fluorescence in situ hybridization and ISS stage. Meanwhile, another 67 risk-, response depth-, and age-matched patients not proceeding to autologous stem cell transplantation were chosen as controls. Our preliminary data indicated that, in the high-risk subgroup, progression-free survival and overall survival were both significantly prolonged after autologous stem cell transplantation (*P* < 0.001 and *P* = 0.015) while, in the intermediate-risk subgroup, neither progression-free survival nor overall survival was prolonged significantly after autologous stem cell transplantation (*P* > 0.05), and in the low-risk subgroup, only progression-free survival was extended significantly (*P* = 0.012) after autologous stem cell transplantation. Multiple variables analysis further indicated that autologous stem cell transplantation and risk stratification were two independent prognostic factors for overall survival. Our results indicated that myeloma patients at different risks all benefit from autologous stem cell transplantation consolidation even in the era of novel agents.

## 1. Introduction

As the second most common hematological malignancy in United States, multiple myeloma (MM) remains incurable though some authors considered it a potentially curable disease [1]. As the key prognostic factors, response depth and genetic heterogeneity play important roles in the survival of myeloma patients [2]. Risk-stratification based assessment is the key of treatment selection. High-dose therapy with autologous stem cell transplantation (HDT-ASCT) is used to be the standard front-line treatment for better responses. However, the development of novel agents, like proteasome inhibitors and immunomodulatory drugs (IMiDs), has significantly improved the survival of MM patients and changed the transplantation scenario in different ways [3]. The necessity of ASCT as an upfront optimal treatment was challenged in the era of new agents, especially based on various risk stratifications. Data in this field is still rare. Two ongoing clinical trials aim to shed some lights on this problem in a few years (NCT01208662, NCT01191060). But so far, there is no conclusion to this question yet. It would be helpful to identify who can benefit most from ASCT and who do not need it any more in the era of novel agents, based on cytogenetic risk stratifications.

Our previous study indicated that MM patients in China are different from those in western countries, not only epidemically (median age 58 versus 70 years old) [4, 5], but also cytogenetically and socially. We also proved that ASCT as a consolidation therapy can achieve better responses in MM patients in China [6]. But there are also large quantities of MM patients eligible for ASCT not proceeding to this treatment due to personal concerns, economic inability, and limited medical resources, since most of guidelines do not take into account the sociology and cost effectiveness before choosing ASCT as upfront treatment [7]. This retrospective study was conducted to evaluate the impact of ASCT as a consolidation therapy on MM patients in different risk stratifications based on the new model of International Myeloma Workshop Consensus (IMWG) [8], defined by fluorescence in situ hybridization (FISH) and International Staging System (ISS) stage, aiming to identify the subgroup of MM patients who can benefit most from ASCT as a consolidation therapy after novel agent inductions.

## 2. Materials and Methods

### 2.1. Patients

From August 2006 to July 2011, 67 consecutive symptomatic MM patients receiving ASCT after induction with bortezomib and/or thalidomide-based combined chemotherapy were enrolled based on risk stratification model of IMWG, including 17 high-risk patients, 24 intermediate-risk patients, and 26 low-risk patients based on FISH and ISS stage. Both* t(4; 14)* and/or 17p13 del were defined as adverse FISH results. High risk refers to ISS II/III and* t(4; 14)*/17p13 del; intermediate risk is defined as ISS III with no adverse FISH or ISS I and* t(4; 14)*/17p13 del; low risk is defined as ISS I/II with no adverse FISH. At the same period, another 67 patients, who did not proceed to ASCT because of personal reasons, were chosen as controls and received continuous bortezomib and/or thalidomide-based chemotherapy as consolidation. Patients were matched based on important prognostic factors according to the following terms: risk stratification, response depth, and age. Firstly, the numbers in different risk subgroups were well matched. Then, the nCR (near complete response)/CR (complete response) rates after inductions were also ensured with no significant differences in each risk subgroup. Besides, the age difference between the control and patient is less than 5 years. In addition to the mentioned factors, there were no significant differences in the sex, M protein class, and other laboratory values of MM patients with or without ASCT (Table 1). At the time of the report, the median follow-up time was 25.6 months (8.1–98.5 months) in all the patients. In high-, intermediate-, and low-risk subgroup, the median follow-up time was 30.6 months (8.1–98.5 months), 23 months (11.5–73 months), and 27.5 months (8.8–72.2 months), respectively. Informed consent was obtained for all the data of enrolled patients.

### 2.2. Induction Regimens and Response Evaluation

All patients received bortezomib and/or thalidomide-based induction therapies and achieved at least PR (partial response) before proceeding to consolidation therapy. Bortezomib was used with 1.75 mg/day on days 1, 4, 8, and 11 or days 1, 4, 7, and 10 of each cycle. Thalidomide was given 75–150 mg per day, with minor adjust according to thalidomide-related adverse event. The median cycles of induction were 6 (4 to 8) in the high-risk group, 4 (3 to 6) in the intermediate-risk group, and 4 (3 to 6) in the low-risk group, with no significant difference between different groups (*P* > 0.05). The efficacy was evaluated every two cycles based on the IMWG criteria [9].

### 2.3. Autologous Stem Cell Transplantation Protocol

Peripheral blood hematopoietic progenitor cells were collected by apheresis after chemomobilization in conjunction with granulocyte colony stimulating factor (G-CSF) (10 *μ*g/kg/day). The method of cell collection was using cyclophosphamide 4 g/m^2^ divided in 2 days. The median number of CD34+ cells was 3.6 × 10^6^ cells/kg. Patients were conditioned with our standard regimen that includes busulfan 0.8 mg/kg IV every six hours on days −7 to −5, etoposide 10 mg/kg IV on days −4 to −3, and cyclophosphamide 60 mg/kg IV on days −3 and −2 [10]. Peripheral blood stem cells were infused on day 0. Patients received G-CSF 5 *μ*g/kg/day from day 5 until neutrophil count more than 1.0 × 10^9^/L was achieved.

### 2.4. FISH

A total of 20 mL of bone marrow was collected from each patient at diagnosis. FISH was performed on CD138+ selected cells.* IgH* probe,* D13S319* probe,* P53* probe, and* 1q21* probe were purchased from GP Medical Technologies (Beijing, China).* CCND1*,* FGFR3*, and* MAF* probes were products of Cytocell (Cambridge, United Kingdom). The reference value was set based on 10 bone marrow samples from patients with no hematologic disease and the normal range was the mean value of the proportion of abnormal cells in total cells ±3 standard deviations.

### 2.5. End Points

The end points of this study were progression-free survival (PFS) and overall survival (OS). PFS was defined as the time from start of treatment to disease progression or death (regardless of cause of death), whichever comes first [11]. OS was defined as the time from diagnosis to death from any cause [12].

### 2.6. Statistical Analysis

All data were expressed as mean ± standard deviation when normally distributed and as median (quartiles) for parameters with nonnormal distribution, unless otherwise specified. Mean comparison among groups and comparison between two groups were performed by one-way ANOVA and chi-square test, respectively. Survival analysis was performed using the Kaplan-Meier method. Differences were considered significant when the *P* value was less than 0.05. Statistical analysis was performed with SPSS software (IBM, version 17.0, New York, NY, USA).

## 3. Results

### 3.1. Responses of MM Patients after Induction Therapy

The nCR/CR rate after induction therapy was 44.8% in ASCT group and 37.3% in non-ASCT group. There were no significant differences in nCR/CR rates between ASCT group and non-ASCT group after induction (*P* = 0.380). In each risk subgroup, there were also no statistic differences. As shown in Table 1, the nCR/CR rates were 41.2% versus 47.1% in high-risk subgroup (*P* = 0.730), 29.2% versus 37.5% in intermediate-risk subgroup (*P* = 0.540), and 42.3% versus 50% (*P* = 0.578) in low-risk subgroup, respectively.

### 3.2. Efficacies of ASCT as Consolidation Therapy

Depth of response was deepened after ASCT. Twelve MM patients who achieved only PR or VGPR after induction therapies further acquired nCR/CR after ASCT. The total nCR/CR rate was elevated from 44.8% to 65.6%. In each risk subgroup, nCR/CR rates were all elevated, from 47.1% to 62.9% in high-risk group, 37.5% to 62.5% in intermediate-risk group, and 50% to 61.5% in low-risk group, respectively.

### 3.3. Survival of Patients after ASCT

**Figure 1:**
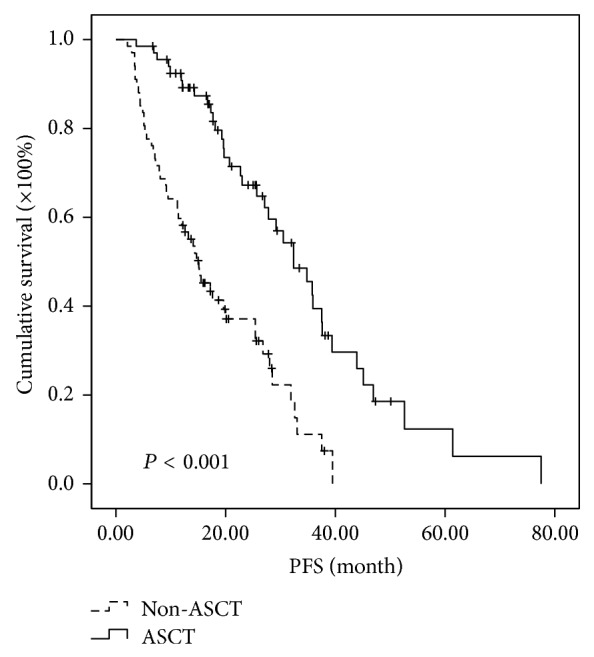
Comparison of PFS in the patients of ASCT group and non-ASCT group regardless of risk stratification. The median PFS was prolonged 17.3 months in ASCT group (32.4 versus 15.1 months, *P* < 0.001).

**Figure 2:**
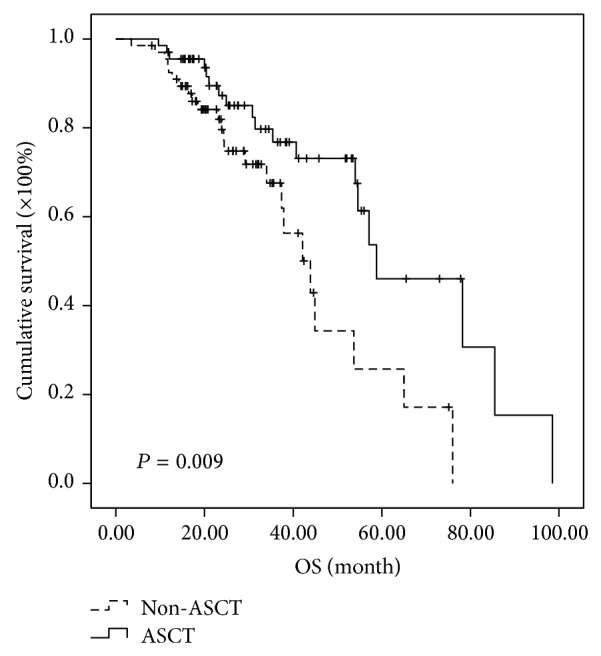
Comparison of OS in the patients of ASCT group and non-ASCT group regardless of risk stratification. The median OS was prolonged 16.7 months in ASCT group (58.8 versus 42.1 months, *P* = 0.009).

The survivals of the 67 patients proceeding to ASCT were compared with the matched controls without ASCT. Comparing to non-ASCT groups, ASCT groups showed longer median PFS (32.4 versus 15.1 months, *P* < 0.001) and OS (58.8 versus 42.1 months, *P* = 0.009) in general (Figures 1 and 2).

**Figure 3:**
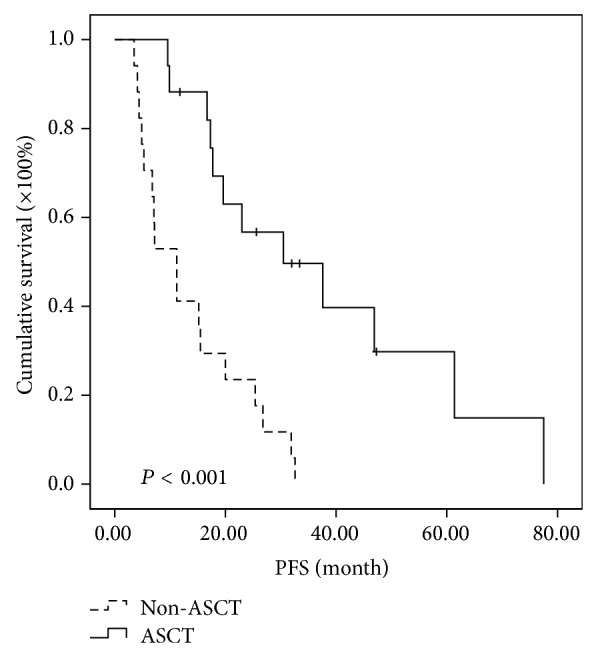
Comparison of PFS in the patients of ASCT group and non-ASCT group in the high-risk subgroup. The median PFS was prolonged 18.3 months in ASCT group (30.5 versus 11.2 months, *P* < 0.001).

**Figure 4:**
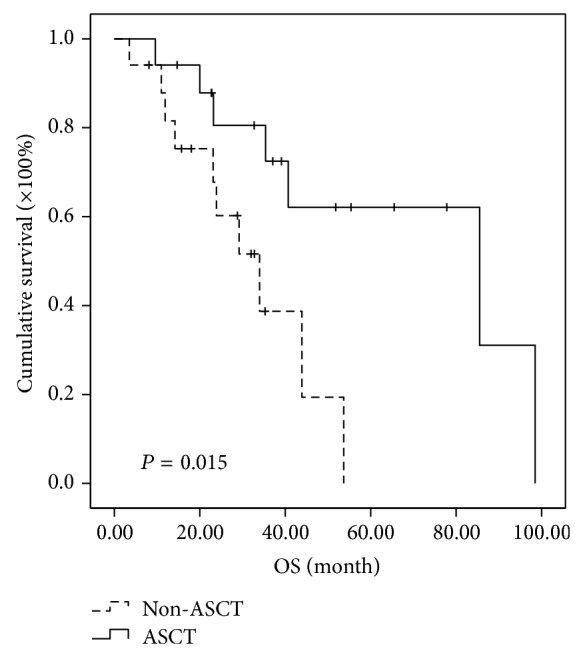
Comparison of OS in the patients of ASCT group and non-ASCT group in the high-risk subgroup. The median OS was prolonged 51.5 months in ASCT group (85.5 versus 34 months, *P* = 0.015).

**Figure 5:**
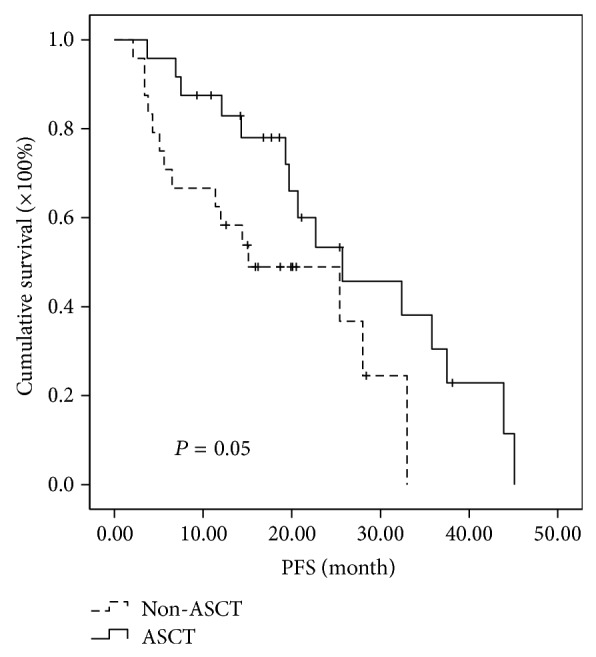
Comparison of PFS in the patients of ASCT group and non-ASCT group in the intermediate-risk subgroup. The median PFS was 25.7 versus 15.1 months with no significant difference (*P* = 0.05).

**Figure 6:**
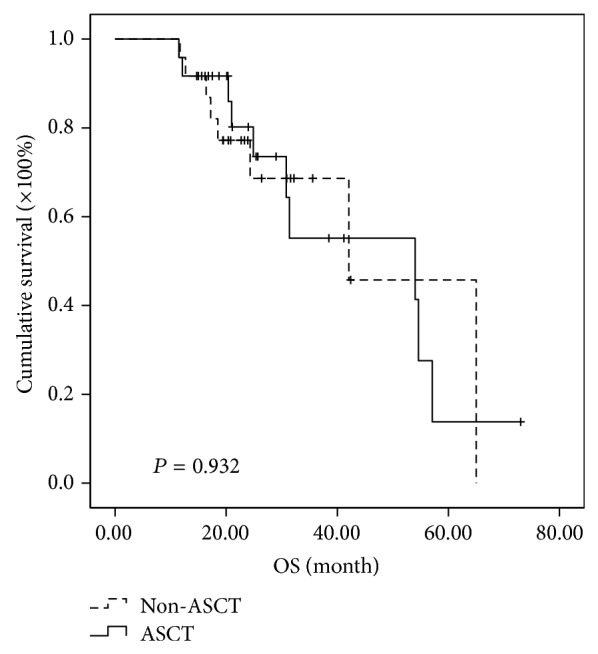
Comparison of OS in the patients of ASCT group and non-ASCT group in the intermediate-risk subgroup. The median OS was 54 versus 42.1 months with no significant difference (*P* = 0.932).

**Figure 7:**
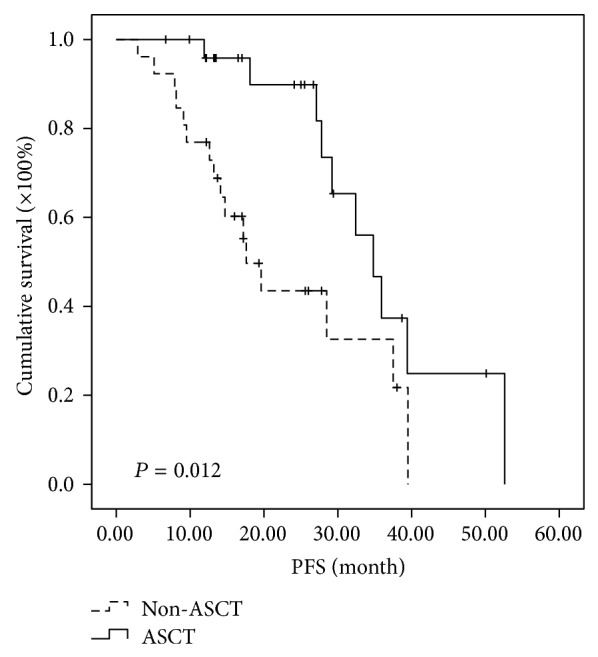
Comparison of PFS in the patients of ASCT group and non-ASCT group in the low-risk subgroup. The median PFS was prolonged 17.2 months in ASCT group (34.8 versus 17.6 months, *P* = 0.012).

**Figure 8:**
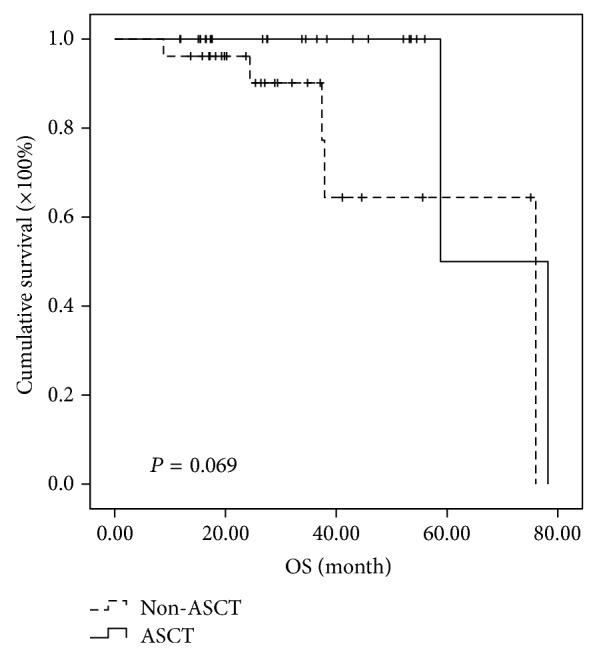
Comparison of PFS in the patients of ASCT group and non-ASCT group in the low-risk subgroup. The median OS was 58.8 versus 76 months with no significant difference (*P* = 0.069).

Further analysis of subgroups indicated that MM patients in different cytogenetic risk categories benefit differently even at the short follow-up time of two years. In the high-risk subgroup, the median PFSs of MM patients in ASCT group and non-ASCT group were 30.5 months and 11.2 months, respectively, while the median OSs were 85.5 months in ASCT and 34.0 months in non-ASCT group. Both PFS and OS were significantly prolonged in high-risk patients receiving ASCT as consolidation (*P* < 0.001 and 0.015, resp.) (Figures 3 and 4). In the intermediate-risk subgroup, it was observed that both PFS and OS have no difference between ASCT and non-ASCT groups. The median PFS was 25.7 versus 15.1 months (*P* = 0.05) and the median OS was 54 versus 42.1 months (*P* = 0.932) (Figures 5 and 6). In the low-risk subgroup, the median PFS was prolonged in ASCT group (34.8 versus 17.6 months, *P* = 0.012) (Figure 7), but the median OS showed no difference (*P* = 0.069) (Figure 8).

### 3.4. Multivariate Prognostic Analysis

Using Cox's proportional hazards regression model, we further performed a multivariate analysis to identify independent factors correlated to OS of all the patients. Variables included age, type of M protein, hemoglobin, albumin, *β*2-microglobulin, ASCT, and risk stratification by FISH. In this analysis, ASCT was an independent prognostic factor associated with prolonged OS (hazard ratio (HR) = 0.396; 95% confidence interval (CI), 0.203–0.775; *P* = 0.007). Compared with low-risk subgroup, both high-risk and intermediate-risk subgroups had significantly higher hazard ratio (HR) for OS (HR = 3.499; 95% CI, 1.494–8.195; *P* = 0.004 for high-risk subgroup and HR = 2.988; 95% CI, 1.250–7.143; *P* = 0.014 for intermediate-risk subgroup). Take it together, only ASCT and risk stratifications were found to be the significant independent prognostic factors in multivariate prognostic analysis.

## 4. Discussion

Here we, retrospectively, investigated 134 MM patients with or without ASCT consolidation therapy after novel agents based inductions, showing that the depth of responses can be further improved in MM patients after transplantation, even under the setting of novel agent inductions, while different risk subgroups benefit differently from ASCT consolidation. In multivariate prognostic analysis, ASCT and risk stratification were found to be independent prognostic factors for OS. Despite considerable advances in the diagnosis and therapy recently, MM remains incurable. High mortality resulting from high-dose and combined chemotherapy was common among patients who were elderly or weak at diagnosis, while dose-reduced therapy may lead to persistence of minimal residual disease (MRD), resulting in disease progression and relapse. The use of ASCT in clinic has improved the survival of MM patients since 1990s, with prolonged PFS time proved in multiple randomized controlled clinical trials (RCTs) [13]. However, the introduction of novel agents, like bortezomib and thalidomide, has changed the scenario of transplantation in different ways and even challenged the necessity of ASCT. Data from different centers showed that the incorporations of these two drugs with ASCT can increase CR rate up to 61% and greatly prolong PFS, ranging from 50% to 69% and 3-year OS ranging from 72% to 85% [14–18], which were similar to our results. Total Therapy 1 (TT1) to Total Therapy 3 (TT3) also demonstrated notable elevation of CR rate and improvement in PFS and OS [19]. Based on these data, induction with novel agents and consolidation with ASCT has been considered the best strategy by far for MM. Our data here confirmed that ASCT can further improve the depth of remission and lead to better survival in MM patients without consideration of risk stratifications. nCR/CR rate was further increased after ASCT in all the different risk subgroups, including cytogenetic high risk myeloma patients, which may also be part of the mechanisms involved in the better survival after ASCT.

However, there are also some controversial evidences challenging the role of ASCT in the era of novel agents, with some trials showing similar CR rates and survival improvements after novel agents based treatments in MM patients not eligible for ASCT. Palumbo et al. [20] reported that, in elderly patients who are not eligible for transplantation, the median PFS was 21.8 months and the median OS was near 4 years for melphalan, prednisone, and thalidomide (MPT) induction therapy group which were similar with patients received ASCT. A meta-analysis of six RCTs also demonstrated that MPT can improve survival more than MP (HR = 0.83 (95% CI, 0.73–0.94), *P* = 0.004), with PFS prolonged by 6.6 months and OS prolonged by 5.4 months [21]. IFM2005-01 phase III trial further proved that the overall response rate (ORR) and the nCR/CR rate were significantly higher with bortezomib plus dexamethasone (VD) than vincristine plus doxorubicin and dexamethasone (VAD), with PFS modestly prolonged after bortezomib based therapy [15]. These results indicated a promising picture that new drugs might achieve a similar quality of remission and survival even without ASCT. It seems that induction therapy with novel agents has been largely recognized nowadays, but the consolidation strategies remain controversial. It is yet unmet to make a conclusion by randomized case controlled clinical data to answer this burning question.

So far as we know, only one prospective clinical trial has been designed to compare the consolidation therapy of ASCT or novel agents in patients after lenalidomide induction therapy [22]. The interim results showed that only PFS was prolonged significantly in patients with ASCT while no significance in OS between the two groups. Since this combination regimen did not include proteasome inhibitors, it might not be representative of the most effective novel combination. Until now, there are very limited data in comparing ASCT and chemotherapy consolidation. In the year of 2013, experts from different centers in Asia like Singapore, Japan, Thailand, South Korea, and China propose that economy and medical resource limitation should be taken into account before making optimal choices for patients in developing countries [7]. Even in China, with the world's largest population, there are still some shortages of medical resources that ASCT is not available in every medical center and not all eligible patients are preceding to ASCT, as guidelines suggested due to personal concerns, inability to pay for medical expense, and limited medical resources. Therefore, it will be helpful to identify the specific patients who can benefit most from ASCT, as well as those who do not need this consolidation after the induction therapy with new agents.

FISH has become the most useful cytogenetic and prognostic marker in MM patient. In this study, we investigated the role of ASCT as consolidation therapy in different risk subgroups according to the latest guideline of risk categories. Reports about the correlation between prognosis and cytogenetic abnormalities have emerged in the past decade. The criteria of mSMART (Mayo Stratification for Myeloma and Risk-adapted therapy) include cytogenetic, GEP (gene-expression profiling), and plasma cell labeling index which could not be performed in every center [23]. A latest risk-stratification model applied by IMWG, combining ISS stage and genetics, was proved to segregate patients into three risk groups [8]. Similar results were seen in another two independent studies from German and MRC (Medical Research Council). Therefore, here we used FISH as a marker to stratify our patients into three risk subgroups (high risk, intermediate risk, and low risk), based on the new criteria approved by multiple famous centers.

Various clinical and laboratory data were proved to be important in the prognosis of MM. Depth of response and cytogenetic stratifications were the most critical ones among them [24, 25]. Patients in this study were case-paired strictly before enrollment. There were no significant differences in clinical features between the patients with or without ASCT, especially the nCR/CR rate before consolidation therapies. Therefore, ASCT was regarded the only difference to impact the PFS and OS of each group, which could be used as the assessment of consolidation therapy with or without ASCT. Our data indicated that ASCT consolidation is beneficial to all the risk subgroups by improving survivals, especially the prolonged PFS and OS in cytogenetic high-risk MM patients, while only PFS was prolonged in lower risk patients, who have favorable cytogenetic markers. At the time of this report, the median OS of various risk subgroups has shown differences even just with a short follow-up time of around two years, suggesting that patients with adverse cytogenetic may need upfront HDT-ASCT even after novel agents based inductions while patients of lower cytogenetic risks do not benefit as much as those with high risk. ASCT as consolidation therapy improves the survival of all the risk stratifications subgroups, but the benefits are different in patients of low- and intermediate-risk groups. These preliminary clinical data is consistent with the interim results of the prospective trial [22]. Multiple variables analysis further indicated that ASCT and risk stratification are two independent prognostic factors for OS. Based on these preliminary data, ASCT consolidation benefits patients with different risks, and cytogenetic risk stratification at diagnosis will help to better predict and design the optimal strategies for different individuals, especially for those who are unable to proceed to ASCT.

This study was carried out in a retrospective manner. Although enrollment of more patients and RCTs is encouraged, our retrospective study provided a preliminary reference for appropriate therapeutic strategies. Further studies are still ongoing to keep follow-up and confirm the impacts of ASCT consolidation in lower risk groups.

## 5. Conclusion

Our study indicated that ASCT could benefit MM patients in general even after novel agents based inductions. But high-risk MM patients defined by cytogenetic stratifications and ISS stage can benefit most from ASCT consolidation and may not need upfront HDT-ASCT even after novel agents based inductions. However, patients of lower cytogenetic risks do not benefit as much as those with high risk, suggesting they could take ASCT as a salvage therapy when relapse occurs.

## Figures and Tables

**Table 1:** Characteristic comparison of MM patients with or without ASCT in three risk subgroups.

	High risk			Intermediate risk			Low risk		
	Non-ASCT	ASCT	*P*	Non-ASCT	ASCT	*P*	Non-ASCT	ASCT	*P*
Number	17	17		24	24		26	26	
Average age (years)	56.0	52.0	0.154	54.0	51.4	0.288	53.5	53.0	0.820
Male/female	11/6	9/8	0.486	16/8	16/8	1.000	18/8	14/12	0.254
IgA class	29.40%	23.50%	1.000	25.00%	20.80%	0.731	19.20%	19.20%	1.000
Average hemoglobin (g/L)	85.5	72.4	0.169	76.9	77.7	0.948	110.4	95.7	0.136
Average serum calcium (mmol/L)	2.69	2.38	0.143	2.62	2.80	0.356	2.44	2.42	0.899
Average creatinine (umol/L)	136.8	188.3	0.456	224.3	160.0	0.493	74.0	90.8	0.189
Average albumin (g/L)	32.5	31.4	0.728	34.7	38.9	0.133	36.5	36.0	0.891
Average *β*2 microglobulin (mg/L)	6.02	6.25	0.884	8.27	6.54	0.374	3.04	3.54	0.33
Median bone marrow plasma cells	49.2%	46.2%	0.803	7.6%	13.8%	0.441	28.8%	35.3%	0.382
nCR/CR rate	41.20%	47.10%	0.730	29.20%	37.50%	0.540	42.30%	50.00%	0.578
